# Measuring the Food Environment: A Systematic Technique for Characterizing Food Stores Using Display Counts

**DOI:** 10.1155/2012/707860

**Published:** 2012-06-04

**Authors:** Cassandra Miller, J. Nicholas Bodor, Donald Rose

**Affiliations:** ^1^Healthy Communities Institute, 2054 University Avenue, 3rd Floor, Berkeley, CA 94704, USA; ^2^School of Public Health and Tropical Medicine, Tulane University, 1440 Canal Street, Suite 2301, New Orleans, LA 70112, USA

## Abstract

Marketing research has documented the influence of in-store characteristics—such as the number and placement of display stands—on consumer purchases of a product. However, little information exists on this topic for key foods of interest to those studying the influence of environmental changes on dietary behavior. This study demonstrates a method for characterizing the food environment by measuring the number of separate displays of fruits, vegetables, and energy-dense snack foods (including chips, candies, and sodas) and their proximity to cash registers in different store types. Observations in New Orleans stores (*N* = 172) in 2007 and 2008 revealed significantly more displays of energy-dense snacks than of fruits and vegetables within all store types, especially supermarkets. Moreover, supermarkets had an average of 20 displays of energy-dense snacks within 1 meter of their cash registers, yet none of them had even a single display of fruits or vegetables near their cash registers. Measures of the number of separate display stands of key foods and their proximity to a cash register can be used by researchers to better characterize food stores and by policymakers to address improvements to the food environment.

## 1. Introduction

Over the past decade, there has been a large increase in research on the neighborhood food environment, with a number of studies documenting associations between the food environment and dietary intake or weight status [1–13]. These and other studies have led to recommendations by scientific panels and policy makers that promote improvements in neighborhood access as a strategy for dealing with the obesity epidemic. For example, the Institute of Medicine listed improving neighborhood access to healthy food as one of the key actions that local governments could take to address the child obesity epidemic [14], and the US President's budget for 2012 included funding for a fresh food financing initiative to improve food access in underserved areas [15].

Despite the tremendous growth, the field is still relatively new, and more research is needed on methods to better-characterize the food environment. One approach to studying it has focused on measures of access to retail food outlets, such as distance to the nearest supermarket [3, 16] or number of supermarkets within a defined area [1, 4, 6, 10]. A second line of research has focused on measures of food within the store. For example, in-store measurements of shelf space have been conducted to study the availability of different types of foods within stores [17], while other studies have looked at inventories of different types of foods or the pricing of foods [8, 18–28].

Marketing research has demonstrated that the in-store environment affects consumer purchasing decisions. It is well known, for example, that the amount of shelf space is an important determinant of sales [29]. Other studies have found that additional display stands influence purchasing behavior [29–32]. For example, in their classic study Wilkinson et al. [32] found that sales increased between 77 and 243% when a brand was displayed in a secondary location. Increasing the number of display stands of a product increases the likelihood that a consumer will encounter it in the store, and thus increases the probability of its purchase, particularly for impulse items. In marketing research that investigated both consumer and in-store characteristics, Inman and coauthors [31] found that additional displays increase unplanned purchases by almost 40% over baseline estimates. The importance of gaining visual attention of the consumer has also been documented in point of purchase studies [33].

Despite the importance of the number of display stands for influencing consumer purchases, and the relative ease in which these data can be collected, very few studies have provided evidence on this variable for foods of particular interest to dietitians and nutritionists [34, 35]. In this study we demonstrate a method for characterizing the food environment by measuring the number of separate displays of various foods and their proximity to a cash register in different types of stores. Because of their documented importance to public health, we focus on fruits, vegetables, and energy-dense snack foods [36, 37].

## 2. Methods

A census of all food stores in the city of New Orleans was developed in the fall of 2007. We began with a commercial list of stores from InfoUSA, which we verified on the ground to assure that listed stores were still open and that new stores were included in the list. Stores were categorized into one of six types: supermarkets, midsize food stores, small food stores, convenience stores (including those attached to a gas station), drug stores, and general merchandise stores. The category of general merchandise stores included local discount retailers and chain discount “dollar” stores that sell a variety of consumer goods in addition to packaged foods. North American Industry Classification System (NAICS) codes and sales data from InfoUSA were used to categorize the stores. Stores with a “supermarket and other grocery store” NAICS code that had annual sales greater than $5 million dollars were classified as supermarkets. Stores with this same code and sales between $1 million and $5 million dollars were classified as midsize food stores, and those with less than $1 million annual sales were categorized as small food stores. Other store types did not rely on sales data for classification and were based directly on the NAICS codes. New stores found on the ground were classified into one of the six categories using information on store characteristics (e.g., number of registers, inventory sold). A 30% random sample of stores was chosen. Additional details regarding development of the store census and sampling have been described previously [38].

In-store observations were taken for 90 stores in 2007 and 113 stores in 2008, with 31 stores observed in both 2007 and 2008. A comparison of data for stores observed in both years did not reveal any significant differences, so these stores were randomly assigned measurements from one of the two years. A total of 172 unique stores were observed, forming the analytic sample for this paper.

Teams of two observers per store collected information on the number of separate displays for five broad categories of fruits and vegetables—fresh fruits, fresh vegetables, canned fruits, canned vegetables and frozen vegetables—and five types of energy-dense snack foods—salty snacks (such as chips and nuts), cookies and crackers, doughnuts and pastries, candies, and carbonated beverages. A continuous linear aisle, or a portion thereof, devoted to a given food category (e.g., fresh fruits), regardless of the number of vertical shelves, was counted as one display, as was a separate island devoted to a given food category. If items for the same food category appeared in two separate linear shelves (e.g., such as in a different aisle, or on opposite facing shelves within the same aisle) they were counted as two separate displays. Displays were counted only once, regardless of the number of specific foods within a given category (e.g., a display aisle of fresh apples, bananas, and/or other fruits was counted as a single display of fresh fruits) nor were they separated by brand (e.g., two or more brands of canned pineapples in a display aisle was counted as a single display). The same method was applied to all food categories, including energy-dense snacks, where brand and type within each snack category were not considered when counting displays. We did not limit our counting of a display to a minimum or maximum length. Observers also recorded whether each separate display was within one meter of a cash register. Interobserver reliability for our method was high with a Pearson correlation value of 0.997 for fruits and vegetables and a value of 0.968 for snacks. Paired *t*-tests showed no significant differences between the mean numbers of displays counted between observers. Supercenters, such as Wal-Mart, were excluded from the analysis, because of the inherent differences in supercenters from other retail food outlets. Additional details on the in-store protocol can be requested from the authors.

Analysis of variance (ANOVA) was used to assess overall differences (*P* < 0.05) in the number of displays by store type. The least significant difference (LSD) test was used post hoc to assess differences in the number of displays for pair-wise combinations of stores. Within a store type (e.g., supermarkets), a paired sample *t*-test was used to assess the difference between the total number of fruit and vegetable displays versus the total number of energy-dense snack food displays. For clarity of presentation, and because of its overriding policy interest, these aggregate food groups (i.e., all fruits and vegetables, all energy-dense snack foods) were used for statistical testing of differences. Data were analyzed using SPSS (version 16.0.1, 2007, SPSS Inc, Chicago, IL, USA). This study is exempt from institutional review as it did not involve human subjects.

## 3. Results

Of the 172 stores that were surveyed, 8 were supermarkets. The most frequently observed stores in the study were convenience stores (*n* = 69) and small stores (*n* = 63).

Almost all stores sold each of the 5 energy-dense snack foods, but the availability of fruits and vegetables differed markedly by store type. Fresh fruits and vegetables were available at all supermarkets and 80% of small stores, but only at 45% of convenience stores and 6% of drug stores (results not shown). Twenty-eight percent of convenience stores did not sell fruits or vegetables of any kind, that is, neither fresh, canned or frozen.

Supermarkets not only had the greatest number of separate displays of fruits and vegetables (mean = 20 ± 10) but also had a much greater number of separate displays of energy-dense snack foods (mean = 80 ± 51) (Table 1). Following supermarkets, midsize stores had the greatest number of separate displays of fruits and vegetables (mean = 9 ± 6) and general merchandise stores had the greatest number of separate displays of energy-dense snack foods (mean = 29 ± 12). Regardless of store type, all stores contained more displays of energy-dense snack foods than of fruits and vegetables.

Not one supermarket in the sample had a single display of any type of fruit or vegetable within a meter of their cash registers (Table 2). Although this was true for most stores in the sample, some midsized stores did have displays of fruits or vegetables that were close to cash registers (mean = 1.0 ± 2.1). In contrast to their stocking practices for fruits and vegetables, supermarkets had many displays of energy-dense snack foods within a meter of their cash registers (mean = 19.9 ± 17.0). Out of the ten food groups studied, the top three items displayed within 1 meter of a cash register for supermarkets were candy, salty snack foods, and carbonated beverages (results not shown). For all other store types the top three items close to registers were candy, salty snack foods, or doughnuts and pastries.

## 4. Discussion

This study has demonstrated a method for characterizing the in-store food environment by counting the number of separate displays of foods and determining their proximity to cash registers. Benchmark results on this information for different store types in a major American city are provided for food groups of importance to those working on obesity and urban food access.

Not surprisingly, supermarkets had more displays of fruits and vegetables than other store types, while drug stores or convenience stores had very few. The availability of these “healthful” foods is consistent with general impressions about supermarkets, as well as with a growing literature that has drawn associations between proximity to supermarkets and positive diet or weight status outcomes [1–4, 6, 7, 39, 40]. No direct comparisons on the number of displays of these foods can be made with previous research, since, to the best of our knowledge, no other studies exist on this topic. But our results are consistent with previous in-store studies showing supermarkets with much greater shelf space of fruits and vegetables than other store types [17].

What is more striking about our results is the sizable number of displays of energy-dense snack foods in supermarkets, particularly the large number of displays of these foods within one meter of store cash registers. Industry research has widely supported that 70–83% of confectionery sales are impulse driven [41]. Recognizing this, most supermarkets and other retail outlets strategically place candy and other items near checkouts. Our findings are consistent with this strategy and with other research on this topic. An observational study of 24 supermarkets in Melbourne, Australia, found that foods displayed at supermarket checkouts were predominantly energy-dense confectionary items [34]. The Food Commission in the United Kingdom surveyed several London supermarkets in 2003 and found all but one of the supermarkets contained confectionery or other snack foods at the checkout [35]. Fruits and vegetables are not usually thought of as impulse items, but supermarkets could certainly experiment with placing snack-size produce—such as individual apples or bananas or prewashed packages of baby carrots—near check-out registers.

While the checkout-counter findings are not surprising, quantitative information about this situation can generate awareness about the problem, and also serve as baseline for measuring progress. To date, most of the policies and programs to address obesity through changes to the food environment have focused on bringing more supermarkets to an area, or on improving the offerings of small stores. For example, financing initiatives have been developed to bring supermarkets to low-income areas [42–47]. In some cases these initiatives can be used by existing small stores in underserved areas, so that they can improve infrastructure to carry more fruits and vegetables. There are also a number of examples of “corner store initiatives,” that is, efforts to convince small store owners to carry healthier foods [12, 48, 49].

However, virtually no work is being done on limiting access to energy-dense snack foods in supermarkets. Given the narrow profit margins in the industry and the importance of stocking decisions to store profits, such work would certainly be an unlikely battle. One viable approach might be to focus on improving the quality of check-out stands, and other aspects of supermarkets, through a voluntary recognition program. Just as the LEED (Leadership in Energy and Environmental Design) designation has sought to encourage green building designs [50], so might a similar program seek to promote characteristics of healthy store designs [14]. Whatever the specific goal that public health nutrition advocates might seek, our study and others like it can provide baseline documentation for efforts to improve in-store aspects of the food environment.

This study is not without limitations. While it takes into account the number of separate displays, it does not consider other factors that might influence consumer purchasing, such as the size or location of separate displays or the prices of foods. Another limitation is the exclusion of whole grains, reduced-fat dairy products, and other important food groups from our study. Our goal here was to focus on a few key food groups that have been linked to obesity and that we could appropriately observe in a larger number of stores. This study was conducted at stores only within New Orleans, so the usual caveat about generalizability from a localized study applies here. Finally, although marketing research has indicated the importance of the number and location of displays, we have no evidence on the impacts of such variables on diet and health outcomes. Additional research is certainly needed in this regard.

## 5. Conclusions

As the field of environmental nutrition expands, more comprehensive assessments of neighborhood food environments are needed. This study demonstrates a useful and relatively simple method for characterizing the in-store environment of retail food outlets by counting the number of separate display stands and their proximity to cash registers for fruits, vegetables, and energy-dense snack foods. Although supermarkets are often thought of as contributing to the healthiness of the food environment, they have many more displays of energy-dense snacks than of fruits and vegetables, particularly at check-out counters. Further research is needed to corroborate these findings and to examine the relationship between in-store display variables and diet and health outcomes.

## Figures and Tables

**Table 1:** Mean number of separate displays, by food group and store type, New Orleans, 2007-2008.

	Supermarket		Midsized		Small store		Conven^1^		Drug store		Genl merch^2^	
Food group	(*n* = 8)		(*n* = 8)		(*n* = 63)		(*n* = 69)		(*n* = 16)		(*n* = 8)	
	Mean	SD	Mean	SD	Mean	SD	Mean	SD	Mean	SD	Mean	SD
All fruits and vegetables^3^	20.1^a^	9.9	9.1^b^	6.5	5.8^c^	3.9	2.7^d^	2.6	3.1^d^	2.7	5.1^cd^	4.4
Fresh fruits and vegetables	12.8	6.8	3.8	2.7	2.4	2.1	0.9	1.3	0.1	0.5	1.1	3.2
Fresh fruits	6.1	2.2	2.0	1.8	1.2	1.1	0.5	0.8	0.1	0.5	0.5	1.4
Fresh vegetables	6.6	4.9	1.8	1.0	1.2	1.2	0.4	0.8	0.0	0.0	0.6	1.8
Can/froz fruits and vegetables	7.4	3.9	5.4	4.2	3.4	2.3	1.8	1.6	2.9	2.4	4.0	2.6
Canned fruits	2.5	1.8	2.4	2.5	1.4	1.0	0.8	0.8	1.9	2.0	2.0	0.8
Canned vegetables	3.5	2.5	2.1	1.7	1.7	1.3	0.9	0.8	1.0	0.6	2.0	2.3
Frozen vegetables	1.4	0.5	0.9	0.4	0.4	0.6	0.1	0.3	0.0	0.0	0.0	0.0

All energy-dense snack foods^3^	79.8^a^	51.3	21.8^bc^	5.7	17.3^c^	6.1	16.8^c^	7.3	24.5^b^	10.7	29.3^b^	12.2
Candy	18.3	13.0	5.4	2.6	3.1	1.6	2.9	1.6	8.8	5.1	9.3	3.5
Salty snack foods	22.9	14.1	6.5	3.3	4.3	2.2	4.4	2.6	5.4	2.9	7.4	4.7
Cookies and crackers	14.4	11.0	3.3	2.1	3.2	1.5	2.9	1.7	4.4	2.4	5.0	1.9
Doughnuts and pastries	12.5	8.8	2.8	1.5	2.4	1.4	2.7	1.6	1.8	1.4	3.0	1.6
Carbonated beverages	11.8	8.1	3.9	1.7	4.3	2.0	3.9	2.0	4.2	1.6	4.6	2.4

^1, 2^“Conven” refers to convenience stores, and “Genl merch” refers to general merchandise stores.

^3^Analysis of variance (ANOVA) was used to study the difference by store type in the total number of displays of fruits and vegetables. The overall ANOVA was significant (*P* < 0.05). The LSD test was used post hoc to test the difference between pairs of stores. Store types sharing a common superscript (e.g., convenience, drug, and general merchandise stores) were not significantly different from each other on number of displays of this food group. A separate ANOVA was done and found to be significant (*P* < 0.05) for differences in energy-dense snack foods by store type. Within each store type, paired sample *t*-tests showed significantly (*P* < 0.05) more energy-dense snack displays than fruit and vegetable displays.

**Table 2:** Mean number of displays within 1 m of a cash register, by food group and store type, New Orleans, 2007-2008.

Store type		All fruits and vegetables		All energy-dense snack foods	
	*N*	Mean	SD	Mean	SD
Supermarkets	8	0.0	0.0	19.9	17.0
Midsized stores	8	1.0	2.1	5.9	3.9
Small stores	63	0.3	0.5	2.8	2.8
Convenience stores	69	0.2	0.5	2.5	1.9
Drug stores	16	0.2	0.4	5.5	3.1
General merchandise stores	8	0.0	0.0	4.9	2.2
